# Factors Associated with Candidiasis in Pemphigus Vulgaris Patients: Results from a Retrospective Study in Two Second-Care Level Hospitals in Mexico

**DOI:** 10.3390/tropicalmed8120521

**Published:** 2023-12-15

**Authors:** Andrés Tirado-Sánchez, Alexandro Bonifaz, María Guadalupe Frías De León

**Affiliations:** 1 Internal Medicine Department, Hospital General de Zona 30, Instituto Mexicano del Seguro Social, Mexico City 08300, Mexico; 2 Laboratorio de Micología, Hospital General de México, Mexico City 06720, Mexico; a_bonifaz@yahoo.com.mx; 3 Unidad de Investigación Biomédica, Hospital Regional de Alta Especialidad de Ixtapaluca, Ixtapaluca 56530, Mexico

**Keywords:** pemphigus vulgaris, neutrophil/lymphocyte ratio, platelet/neutrophil ratio, corticosteroids, candidiasis, candidemia

## Abstract

Background: Infections are a major cause of morbidity and mortality in patients with pemphigus vulgaris (PV). One of the most common infections in these patients is candidiasis. This is probably due to the use of systemic immunosuppressants, including oral and intravenous corticosteroids, mainly in megadoses (pulse therapy), although it is unknown if there are other associated factors, in addition to immunosuppressive treatment. We determine the factors associated with candidiasis in PV patients in two second-care level hospitals in Mexico. Methods: We reviewed 100 cases with PV. Cases were randomly selected from the databases of two second-care level hospitals between January 2010 and December 2019 (10 years). The primary endpoint was the incidence of candidiasis in patients with PV. Results: One hundred patients with PV were enrolled in this retrospective study. Candidiasis was observed in 79 patients (79%). A maximum corticosteroid dose of 55 mg/day during the last year (*p* = 0.001) and a higher neutrophil/lymphocyte ratio were associated with candidiasis in patients with PV (*p* = 0.001). Conclusion: Risk factors favoring candidiasis in patients with PV are not only related to the use of corticosteroids, but also to demographic factors, the activity of the disease, and the systemic inflammation associated with autoimmunity.

## 1. Introduction

Pemphigus vulgaris (PV) is an autoimmune disease associated with significant morbidity and mortality, and treatment with high-dose corticosteroids may increase the risk of infection, including candidiasis [1,2]. The annual PV incidence varies from 0.76 to 16.1 cases per million, according to several studies [1,2]. Systemic steroids can cause several physical and mental health problems, including decreased bone density, cataracts, water and electrolyte imbalances, peptic ulcers, pancreatic inflammation, mental illness, diabetes, Cushing’s syndrome, and opportunistic infections, including candidiasis. Infections are the most frequent complications of patients with pemphigus and account for 34.3–55.5% of all deaths [1], and therefore, early identification can lead to more effective and timely therapy, with a better prognostic value.

Pathogens such as *Candida* usually coexist with other microorganisms in the skin microbiota and do not cause disease, but immunosuppressive conditions can promote their excessive growth, resulting in clinical manifestations. The *Candida* species may contribute to the development of mucocutaneous infections, endocarditis, intravascular catheter infections, osteoarticular infections, and meningitis, which can ultimately be fatal if inadequately treated; the prompt and effective treatment of such infections is essential to prevent serious complications [1].

Candidiasis may be more common in immunocompromised individuals, while in immunocompetent patients, the use of broad-spectrum antibiotics, immunosuppressive agents, chemotherapy, intravenous catheters, parenteral nutrition, surgery, malignancy, diabetes, and other factors may contribute to the incidence of candidiasis. Candidiasis is prevalent in patients with PV [1].

The treatment of PV often requires high-dose systemic steroids and adjuvant steroid-sparing drugs, such as azathioprine or mycophenolate mofetil [3], which are valuable for effectively reducing recurrence rates when systemic steroids are tapered off or withdrawn [4]. High-dose steroids in pulse therapy or rituximab are used in cases of an inadequate response to standard treatment or when in relapse (defined as the appearance of at least three new lesions within 1 month that do not resolve within 1 week without treatment, or the worsening of pre-existing lesions in a patient with a controlled disease) [5,6].

Remission during treatment is characterized by the absence of new or existing lesions while the patient is receiving minimal therapy [6]. The duration of remission varies among patients, and in some cases, remission lasts longer. Patients may develop infections that complicate the disease course, regardless of the disease activity, relapse, or remission [7,8]. The early detection of these infections can lead to effective treatment regimens.

Candidiasis incidence varies with steroid dose and duration of use, but the specific steroid dose required to induce candidiasis remains unclear. Previous studies in patients with systemic lupus erythematosus found an increased risk of candidiasis with high-dose corticosteroids: the maximum dose was 24 mg (range 4–250 mg) and the cumulative dose over 3 months was 1.18 g (range 360–4320 mg). These results showed that the risk of candidiasis is related to disease activity, which results in higher systemic steroid requirements; however, other factors may be involved in candidiasis development. This study aims to evaluate the factors associated with candidiasis in patients with PV at different stages of disease progression.

## 2. Material and Methods

In this study, the medical records of 100 patients with a diagnosis of pemphigus vulgaris were retrospectively analyzed. The patients were randomly selected from two second-care level hospitals over a period of 10 years (between January 2010 and December 2019). The diagnosis of PV was confirmed by clinical, histopathologic, and immunologic criteria, in accordance with an international expert panel [6]. Epidemiological data on demographics, disease characteristics indicating disease activity and severity, comorbidities including diabetes, hypertension, and cardiovascular and cerebrovascular disease, indicated treatments (oral steroid dose, adjuvant, and steroid pulse), biochemical parameters, candidiasis history, candidiasis treatment, and PV prognosis or outcome (death) were meticulously recorded from medical files and the hospital electronic medical record. Because of the retrospective study design, we were unable to assess the severity of mucosal involvement, so we assessed only cutaneous involvement, which we arbitrarily categorized as mild (<5%), moderate (5% to 15%), or severe (>15%).

The exclusion criteria were incomplete clinical records or patients with less than three clinical follow-up visits; also, patients with autoimmune diseases different from PV or non-*Candida* mycoses were excluded.

Patients were categorized into those who developed candidiasis during the follow-up period and those who did not develop candidiasis. All forms of candidiasis, including cutaneous, oral, vaginal, and blood, were recorded.

Potential risk factors for candidiasis, as well as factors associated with pemphigus vulgaris or immunosuppressive therapy that may contribute to the incidence of candidiasis, were documented in the present study. The risk factors included the presence of sepsis (previous infections in different sites were documented), immunosuppressive factors, and the use of corticosteroids or other immunosuppressants. Biochemistry and serology markers associated with systemic inflammation were also calculated, which were mainly the neutrophil/lymphocyte ratio (NLR) and the platelet/lymphocyte ratio (PLR).

As this was a retrospective study with no patient data and no interventions, ethics committee approval was not necessary, and only the study completion was reported.

### Statistical Analysis

Statistical analysis was performed using SPSS version 24 (IBM, Armonk, NY, USA) for Windows. Descriptive and two-tailed comparative tests, including Chi-squared and Student’s *t*-tests, were performed for categorical and numerical variables, respectively, to compare patients with and without candidiasis, and multivariate regression analysis was performed to determine the association between various study factors and candidiasis development. Only significant variables in the univariate logistic regression analysis were considered for multivariate analysis. Statistical significance was defined as a *p*-value being less than 0.05.

## 3. Results

### 3.1. Demographic, Clinical, and Treatment Characteristics

Patient demographic and clinical data are shown in Table 1. The female to male ratio was 1.6:1, with 62 (62%) female and 38 (38%) male patients included in the study. The mean age of the patients was 47.94 ± 10.42 years (the range was from 28 to 68 years). The majority of the patients selected were either in the active phase of the disease or in the remission phase. All patients with active disease or relapse (60 cases, 60%) had mucosal (oral and/or genital) and cutaneous involvement (mucocutaneous PV).

### 3.2. Candida Species Distribution

Table 2 presents a list of etiological agents associated with candidiasis in PV patients. Candidiasis in PV is commonly associated with *C. albicans*, although in 32% of the cases (27 patients), it was not possible to isolate the causative agent. Systemic treatment was used in the majority of cases to treat candidiasis, due to the immunosuppression by the treatment. The mucosal ulceration caused by PV results in severe pain and makes topical treatment difficult, thus necessitating systemic approaches. The most common systemic treatment used was fluconazole (34%), followed by itraconazole (32%).

### 3.3. Comparative Characteristics in Patients with Pemphigus Vulgaris with and without Candidiasis

The comparative characteristics of patients experiencing PV with and without candidiasis are presented in Table 3. Demographic variables and comorbidities did not significantly differ between the two groups. However, the NLR and the PLR, two variables associated with systemic inflammation, showed substantial differences between the two groups (*p* = 0.001 and *p* = 0.002, respectively). Patients who developed candidiasis received a higher dose of corticosteroids, which is indicative of the greater severity of the disease and the consequent need for high doses of corticosteroids, which are typically administered in megadoses (pulse therapy) (Table 3).

### 3.4. Underlying Comorbidities

Comorbidities were detected in 46% of the PV cases, with thirty-seven patients having one or more candidiasis events. In those without a history of candidiasis, nine patients had comorbidities, but no significant difference was found. Hypertension, diabetes mellitus, chronic renal insufficiency, and coronary artery disease were the most common comorbidities in both groups. Malignancy was observed in only ten cases. Table 3 shows the distribution of comorbidities among the patients studied.

The death rates were significantly higher in patients with candidiasis than in those without candidiasis (14% vs. 5%, *p* = 0.025). Candidiasis was found to be more common as PV severity increased (related to the body surface area affected) (OR = 3.18; IC^95%^ 1.25–4.2; *p* = 0.01), and it was also associated with an increased need for systemic steroids and other immunosuppressive agents, particularly azathioprine, resulting in a fatal outcome.

### 3.5. Identified Risk Factors

The risk factors for candidiasis in patients with PV were determined through multivariate analysis, as presented in Table 4. Several factors may influence the development and predisposition to candidiasis in patients with PV, including an age of 65 years or older, being of the female sex, having sepsis, experiencing disease relapse or activity, the use of pulse therapy (high-dose steroids), and an elevated NLR ≥ 2 and PLR ≥ 150.

### 3.6. Source of Candidiasis

We found that thirty-six out of 100 cases had candidiasis of the oral mucosa, and, while thirty-two of these (89%) were cases with active PV, only four cases (11%) were relapses of the disease, and none were remission cases. *Candida albicans* was isolated from the majority of cases (47 percent), and the most common sites were oral (36 percent) and genital (35 percent), followed by skin (29 percent) and urine (15 percent) samples. Candidemia was present in fourteen patients, eight of whom (57 percent) had a history of *Candida* isolations from two or more sites. The genital mucosa fourteen cases) and urine (eight cases) were the most common sites of isolation in patients with candidemia. *Candida glabrata* was the most common non-albicans *Candida* species isolated from the genital mucosal and urinary tracts in patients with candidemia, with four and three cases, respectively. None of the specimens were tested for sensitivity.

### 3.7. Mortality in Pemphigus Vulgaris with Candidiasis

A total of twelve fatal cases were observed in this study, eleven of which occurred in patients with a history of candidiasis. Candidemia was present in five of these cases (45%), with *C. albicans* as the sole cause of isolation. *C. albicans* (eight cases), *C. glabrata* (three cases), *C. parapsilosis* (three cases), and *C. tropicalis* (two cases) were the causative agents of candidiasis and candidemia in these patients.

All cases of candidiasis in the patients with pemphigus vulgaris who died were treated with intravenous fluconazole. Voriconazole followed by fluconazole was used in only two cases.

## 4. Discussion

This study adds to our knowledge of the prevalence of candidiasis in patients with PV. In addition, it provides new data on the risk factors that influence the presence of candidiasis and candidemia in this patient population.

The incidence of candidiasis and candidemia could be modified according to the characteristics of each hospital and patient group. However, it is possible that patients with PV are at high risk of developing candidiasis and subsequent candidemia due to the numerous risk factors they present.

Currently, in patients with risk factors for candidemia, some treatment guidelines suggest the initiation of empiric therapy. However, it is common for patients with PV to have other factors that may influence the risk of candidemia, such as other immunosuppressive therapies, comorbid conditions, sepsis, the empiric use of broad-spectrum antibiotics, malignancy, intravenous catheterization, urinary catheterization, and intensive care unit admission.

More than 40 species have been identified as responsible for human candidiasis [9]. Of these, at least 17 are capable of causing invasive disease [10]. However, some are more likely to cause superficial infections than invasive disease [11]. The most common species were *Candida albicans*, *Candida glabrata*, *Candida tropicalis*, *Candida parapsilosis*, and *Candida krusei*, with *Candida albicans* causing 81.3% of cases [12]. Our study shows that this pathogen caused only 47% of cases, which is lower than that reported in the literature. In 32% of cases, it was not possible to isolate the yeast, resulting in unidentified species. Therefore, it can be assumed that the proportion of *C. albicans* is higher in this group, similar to previous research. In another study, it was found that 57.14% of immunocompromised individuals had an isolation of *C. albicans*, a prevalence closer to that observed in the present study [13]. *C. (Nakaseomyces) glabrata* was the second most common etiologic agent, representing 9% of cases, particularly in women aged 65 years and older, in agreement with the existing literature indicating that *C. glabrata* occurs in older women with hematologic malignancies [14]. Unfortunately, molecular techniques to differentiate the *Nakaseomyces* species from the closely related species *Candida nivariensis* and *Candida bracarensis*, which share similar niches in humans, were not used, due to the retrospective nature of the study [15]. The prevalence of *C. albicans* and non-albicans *Candida* species in PV patients are high.

The median age of the patients participating in the study was 47 years, which is within the range reported in the literature [16]. A female predominance was observed in this study, as in previous epidemiologic studies of PV [17].

Several risk factors have been identified for both candidiasis and candidemia, including the use of broad-spectrum antibiotics in addition to topical and systemic antifungals, which can alter and damage the microbiota, promoting *Candida* colonization [18,19].

In immunocompromised patients and among those receiving immunosuppressive therapies, candidiasis is a common complication [20]. Also, it is a common complication among individuals with PV, a rare autoimmune disease of unclear etiology. This is due to the increased need for corticosteroids and other immunosuppressive therapies to manage the disease’s activity, the increased susceptibility to infection, due to skin damage, and the development of severe comorbidities that further compromise the immune system [21,22].

A recent study of 25 PV patients showed that 20% of patients had oral candidiasis. *C. albicans* was predominant in 22 out of 25 isolates, with 100% being susceptible to amphotericin and econazole, and 96% being susceptible to fluconazole and posaconazole [1]. In our study, antifungal susceptibility testing was not carried out, as it is not a routine test in either of the two hospitals from which the patients were collected. However, all cases were reported to have responded satisfactorily to the treatments given or failed to progress to more severe clinical conditions, except in the cases that developed candidemia.

In previous studies, *Candida* species have been found to be sufficiently susceptible to treatments such as caspofungin, amphotericin, flucytosine, voriconazole, and micafungin. Many of these treatments are not available in the hospitals where the study was conducted. Candidiasis with or without candidemia is usually initiated with fluconazole, although resistance rates of between 6 and 25%, and even higher, have been reported in the literature for non-albicans *Candida* species, most notably *C. glabrata* and *C. krusei*, requiring even higher doses of systemic antifungals than cases associated with *C. albicans*. Other antifungal regimens, such as echinocandins, are recommended for candidemia caused by non-albicans *Candida* species [18].

Although topical steroids are not the primary treatment for PV, they are often used to control lesion severity and to reduce the need for systemic steroids, which may contribute to the occurrence of opportunistic infections such as candidiasis. In a study conducted by Lozada et al., which included 55 patients with oral vesicular erosive disease, the effect of adding clobetasol propionate to fluocinolone was observed, and 13 patients (24%) were found to have candidiasis at the end of treatment (28 days) [21].

Although there was no difference in the use of mycophenolate mofetil in patients with PV who developed candidiasis compared to those who did not develop candidiasis, it is interesting to note that, in the multivariate analysis, mycophenolate use exerted a protective effect against developing candidiasis. Mycophenolate mofetil is an immunosuppressive agent that is used in the avoidance of transplant rejection and in the treatment of autoimmune diseases such as PV. Mycophenolate is a 2-morpholinoethyl ester of mycophenolic acid, the latter being the active form of the mycophenolate mofetil [23].

*C. albicans* has been shown to be sensitive to mycophenolate when cultured in vitro and to inhibit inosine monophosphate dehydrogenase, the therapeutic target of mycophenolate mofetil [23]. However, previous studies have observed the resistance of *C. albicans* to mycophenolic acid, which probably did not allow for the observation of clear differences between groups or for a significant reduction in the risk of infection [24].

Thirty patients were treated using rituximab, and no differences between the two groups were observed. Rituximab is a chimeric murine and human monoclonal antibody that selectively targets CD20-positive B-lymphocytes, causing complement-mediated B-lymphocyte lysis, cell cytotoxicity, and the induction of apoptosis. It is an effective treatment for PV, but it can cause opportunistic infections, which do not require treatment interruption but rather infection management [25], and it does not significantly increase the risk of candidiasis, as observed in the study.

Rather than being a consequence of immunosuppression associated with systemic steroids, it is likely that the increased incidence of *Candida* plays a greater role in the development of autoimmunity in PV. Superantigens have been shown to induce the polyclonal activation of memory T-cells (CD45RO+), resulting in a dysregulated response that may lead to opportunistic infections, primarily *Candida*; this increased response may be the cause of autoimmune activity in PV [26].

In this study, we found that 79 out of 100 patients with PV at different stages of severity and activity developed candidiasis at some point during the disease, but the underlying factor causing this predisposition has not been elucidated, as it is uncertain whether it is due to the use of immunosuppressive drugs or to other factors.

Corticosteroids are the mainstay of treatment in PV. Such therapies are aimed at suppressing disease activity, inevitably depleting T cells by inhibiting cytokine transcription (mainly proinflammatory cytokines). In this study, we analyzed several variables related to the steroid requirements for the control of PV activity, such as the maximum steroid dose, the cumulative dose, and the requirements for megadoses of steroids (pulsed therapy), which cause the suppression of the immune function. Suggested mechanisms to explain the effect of steroids on the development of candidiasis include the reduction in T cells, which affects the immunosuppression that promotes candidiasis, such as that which occurs in patients with HIV infection, as well as the inhibition of other immune system cells, such as macrophages and B cells [27].

Age and gender were associated with a higher risk of developing candidiasis during the course of the disease, regardless of the use of steroids, but not for comorbid conditions such as diabetes and hypertension.

In addition, we found that the development of candidiasis may be independently influenced by the activity and relapse of PV, as well as the onset of sepsis. The damaged skin experienced in PV may facilitate bacterial and fungal invasion, increasing the risk of infection, which is exacerbated by the increased need for corticosteroids, often administered via pulse therapy, to induce immunosuppression. These factors collectively contribute to an increased infection frequency and severity.

Candidiasis is strongly associated with immunosuppressed patients, and in this study we observed that patients with a higher need for immunosuppressant therapy with systemic steroids had a higher incidence of candidiasis. Consequently, the changes observed in markers of systemic inflammation, such as the NLR, correlated not only with PV activity, but also with a predisposition to candidiasis and candidemia [28].

Two markers of systemic inflammation, the NLR and the PLR, which are correlated with the activity and severity of PV, were found to be elevated in patients with candidiasis, and elevated levels of these markers were, in turn, associated with a high risk of developing candidiasis [16]. The elevated levels of the NLR presented in this study may be attributed to an activated immune response to PV activity or relapse, as well as to a bacterial infection resulting in increased neutrophils and decreased lymphocytes, the latter being further decreased in inflammatory conditions and with megadoses of corticosteroids [16,29].

Additionally, the PLR has been proposed as another marker of systemic inflammation, although it is less studied than the NLR [30]. Platelets play a critical role in inflammatory and tissue repair processes, as supported by a growing body of evidence [31]. Platelets closely collaborate with all types of leukocytes, releasing chemotactic substances that expedite leukocyte adhesion to the endothelial surface and their subsequent extravasation [31]. These substances may also have both stimulatory and inhibitory effects on leukocyte inflammatory responses [32]. Chronic low-grade inflammation may increase the PLR and the risk of coronary heart disease, solid tumors, and the autoimmunity associated with them. Elevated levels of inflammatory markers may contribute to a pro-inflammatory environment that is conducive to the growth of bacterial and fungal infections [33,34].

Several studies have described an increase in the NLR in diverse inflammatory diseases, along with an association between its levels and disease activity, suggesting a prognostic role [35,36,37]. This increase in NLR results from a rise in neutrophil numbers in the early stages of acute inflammation, while lymphocyte numbers are relatively low. At later stages, there is subsequent activation and lymphocyte proliferation in response to chronic inflammation, whereas the number of neutrophils is relatively low [38].

We have observed that elevated levels of the NLR and the PLR may increase the likelihood of acquiring candidiasis and candidemia, possibly due to an increased severity of the disease and a greater need for immunosuppressive drugs, particularly high doses of corticosteroids.

The major strength of our research is that the selection of participants was entirely random, from the entire pool of patients with PV from two hospital centers over a considerable period of time, allowing us to avoid selection bias. In addition, because all patients with PV received identical oral treatment and follow-up protocols over time, the patient cohort in this study was likely homogeneous.

However, a major limitation of our study is in its retrospective design, which, in view of the low prevalence of the disease, is an obstacle to the undertaking of prospective studies. However, we believe that the patient sample is significant and has sufficient power to draw valid conclusions. To reduce potential selection bias in single-center retrospective studies, patients from two second-care level hospitals were randomly selected. To confirm our findings, these results may be complemented by future multicenter studies.

## 5. Conclusions

Patients with PV were found to have a higher frequency of candidiasis and a lower proportion of candidemia.

The results suggest that this incidence is related to disease severity and activity, as well as the total steroid dose required; however, other factors such as age, sex, the development of sepsis, previous antibiotic and antifungal use, and the severity of systemic inflammation may contribute to the increased incidence of candidiasis in this patient population.

Candidiasis in autoimmune patients undergoing immunosuppressive therapy has been linked to a number of risk factors, each of which is controversial in its own right. Finally, immunology studies report that T lymphocyte depression directly influences the development of candidiasis, although, as observed in the results of our study, there are other factors that may contribute to the development of infection in varying degrees.

Clinicians should be aware that the risk of candidiasis in patients with PV may be increased by factors other than corticosteroid use. Monitoring the maximum corticosteroid dose and other serological markers in susceptible patients may help to prevent candidiasis. Patients should also be informed about the risk of candidiasis due to the use of corticosteroids or other factors unrelated to the immunosuppressive effects of the therapies. To investigate these risk factors for candidiasis and to draw more robust conclusions, further studies are needed.

## Figures and Tables

**Table 1:** Demographic and clinical characteristics of PV patients.

Patient Characteristics	Distribution (n = 100)
Age, years, mean ± SD (range)	47.94 ± 10.42 (28–68)
Male/female, n (%)	38 (38)/62 (62)
Patients who relapsed, n (%)	20 (20)
Patients with active disease *	40 (40)
Patients in remission, n (%)	40 (40)

* Active disease: this was defined as the appearance of more than five blisters per week and the Nikolsky or Asboe-Hansen sign.

**Table 2:** *Candida* species and antifungal agents in PV patients with candidiasis.

Characteristic	Distribution, n = 79 (%)
*Candida* species (85 isolates in 79 patients)	
*C. albicans*	40 (47)
*C. glabrata*	8 (9)
*C. tropicalis*	4 (5)
*C. parapsilosis*	4 (5)
*C. krusei*	2 (2)
*Candida* sp.	27 (32)
Antifungal agents (n = 79)	
Topical antifulgal	20 (25)
Fluconazole	27 (34)
Itraconazole	25 (32)
Voriconazole	7 (9)

**Table 3:** Comparative characteristics of PV patients with and without candidiasis.

Variables	All Patients (n = 100)	Candidiasis		*p*-Value
		Absent (n = 21)	Present (n = 79)	
Age, mean ± SD	47.94 ± 10.42	50.38 ± 10.75	46.17 ± 9.99	NS
Gender, female	62 (62)	14 (67)	50 (63)	NS
Any comorbidity	46 (46)	9 (43)	37 (47)	NS
Diabetes	28 (28)	8 (38)	20 (25)	NS
Hypertension	37 (37)	8 (38)	29 (37)	NS
Coronary artery disease	12 (12)	4 (19)	8 (10)	NS
Chronic renal disease	15 (15)	7 (33)	8 (10)	NS
Malignancy	10 (10)	3 (14)	7 (9)	NS
Cerebrovascular events	5 (5)	1 (5)	4 (5)	NS
NLR, mean ± SD	3.6 ± 2.9	2.68 ± 0.91	4.16 ± 1.14	0.001
PLR, mean ± SD	183.23 ± 89.4	155.25 ± 49.7	237.55 ± 63.16	0.002
PNR, mean ± SD	60.25 ± 18.35	59.78 ± 12.84	57.38 ± 5.17	NS
Sepsis	24 (24)	5 (24)	19 (24)	NS
*Candida* spp. in oral cavity	36 (36)		36 (45)	
*Candida* spp. in genital mucosae	35 (35)		35 (44)	
*Candida* spp. in skin	29 (29)		29 (37)	
*Candida* spp. in urine sample	15 (15)		15 (19)	
Candidemia	14 (14)		14 (18)	
Prior antibiotic use, including extended spectrum antibiotics	39 (39)	5 (24)	34 (43)	0.015
Prior antifungal agent use	27 (27)	3 (14)	24 (30)	0.019
Pulse corticosteroid therapy	46 (46)	3 (14)	43 (54)	0.001
Maximum dose of corticosteroid (mg/day)	55.32 ± 18.49	51.9 ± 5.58	67.67 ± 12.09	0.012
Latest dose of corticosteroid (mg/day)	15.28 ± 8.33	6.07 ± 2.02	17.83 ± 7.6	0.001
3 months’ cumulative dose of corticosteroid (mg)	3249 ± 1523	2602.1 ± 482.72	3602.17 ± 886.26	0.012
Rituximab	30 (30)	6 (28)	24 (30)	NS
Azathioprine	88 (88)	18 (86)	70 (89)	NS
Mofetil mycofenolate	38 (38)	7 (33)	31 (39)	NS
Overall death	12 (12)	1 (5)	11 (14)	0.025

SD = standard deviation; NLR = neutrophil/lymphocyte ratio; PLR = platelet/lymphocyte ratio; PNR = platelet/neutrophil ratio.

**Table 4:** Multivariate analysis of the risk factors for candidiasis in PV patients.

Variable	OR	^95%^CI	*p*-Value
**Age (≥65 years)**	**1.5**	**0.92**–**3.23**	**0.038**
**Gender (female)**	**1.45**	**1.23**–**3.38**	**0.025**
Comorbidity	0.58	0.26–1.38	0.078
Diabetes mellitus	0.78	0.4–1.86	0.123
Hypertension	0.85	0.71–1.89	0.14
Coronary artery disease	0.45	0.28–1.39	0.33
Chronic renal disease	0.38	0.27–2.22	0.146
Malignancy	1.28	0.42–3.84	0.066
Cerebrovascular event	1.65	0.19–4.58	0.079
**Sepsis**	**2.78**	**1.25**–**7.36**	**0.025**
**PV relapse**	**3.25**	**1.89**–**7.23**	**0.015**
**Active PV**	**2.89**	**1.15**–**4.35**	**0.028**
**PV severity (≥15% BSA)**	**3.18**	**1.25**–**4.2**	**0.01**
**Maximum corticosteroid dose (≥50 mg/day)**	**4.85**	**1.87**–**6.43**	**0.001**
**Pulse corticosteroid therapy**	**3.59**	**2.23**–**4.89**	**0.002**
**Prior antibiotic use**	**6.48**	**1.12**–**7.23**	**0.042**
**Prior antifungal use**	**3.45**	**1.22**–**3.33**	**0.035**
Mycophenolate mofetil	0.65	0.33–0.98	0.04 *
Rituximab	1.1	0.45–1.23	0.13
**NLR (≥2)**	**3.38**	**1.27**–**4.59**	**0.001**
**PLR (≥150)**	**2.85**	**1.84**–**3.59**	**0.001**
PNR (≥50)	1.12	0.24–2.23	0.057

OR = odds ratio; BSA = body surface area affected; PV = pemphigus vulgaris; NLR = neutrophil/Lymphocyte ratio; PLR = platelet/Lymphocyte ratio; PNR = platelet/Neutrophil ratio; * protective effect. Significant parameters are highlighted in bold.

## Data Availability

Data supporting the reported results can be requested from corresponding author.
